# LATE-ONSET SELF-HEALING LANGERHANS CELL HISTIOCYTOSIS: REPORT OF A VERY RARE ENTITY

**DOI:** 10.1590/1984-0462/;2017;35;1;00015

**Published:** 2017

**Authors:** Fatma Sule Afsar, Malik Ergin, Gulcihan Ozek, Canan Vergin, Ali Karakuzu, Sila Seremet

**Affiliations:** a Ataturk Research and Training Hospital, Esmirna, Turquia.; b Dr. Behcet Uz Children’s Hospital, Esmirna, Turquia.; c Katip Celebi University, Faculdade de Medicina, Esmirna, Turquia.

**Keywords:** Infant, Histiocytosis, Langerhans-cell, Self-healing

## Abstract

**Objective:**

To report a case of late-onset self-healing Langerhans cell histiocytosis.

**Case description:**

A 4½-month-old female patient presenting with an eythematopurpuric eruption underwent a skin biopsy for histopathology and was first diagnosed with isolated cutaneous Langerhans cell histiocytosis. Her lesions regressed within a few months and she was retrospectively diagnosed with late-onset self-healing Langerhans cell histiocytosis after being without skin or systemic involvement in a follow-up four years later.

**Comments:**

Self-healing Langerhans cell histiocytosis, which is characterized by clonal proliferation of Langerhans cells and presents with cutaneous lesions, is a rare self-limited variant of histiocytosis and can only be diagnosed retrospectively, after the patient remains free from systemic involvement for several years. Although it presents at birth or during the neonatal period, only a few cases of its late-onset type regarding the age of onset have been reported. Purpuric lesions that appear after the neonatal period serve as a clue for late-onset self-healing Langerhans cell histiocytosis and the patients should be monitored regularly for systemic involvement if the diagnosis is confirmed by a cutaneous biopsy.

## RESUMO

**Objetivo:**

Relatar um caso de histiocitose de células de Langerhans autolimitada e de início tardio.

**Descrição do caso:**

Paciente com 4 meses e meio de idade do sexo feminino, apresentando uma erupção cutânea eritematosa purpúrea, foi submetida a uma biópsia de pele, sendo diagnosticada com histiocitose de células de Langerhans cutânea isolada. As lesões regrediram em poucos meses e ela foi diagnosticada, retrospectivamente, com histiocitose de células de Langerhans autolimitada e de início tardio, após não apresentar nenhum envolvimento cutâneo ou sistêmico durante um seguimento de quatro anos.

**Comentários:**

A histiocitose de células de Langerhans autolimitada caracteriza-se pela proliferação clonal das células de Langerhans e apresenta-se com lesões cutâneas, sendo uma variante autolimitada rara de histiocitose. A doença só pode ser diagnosticada de forma retrospectiva, após o paciente não apresentar nenhum envolvimento sistêmico durante vários anos. Embora existam casos de manifestações ao nascimento ou durante o período neonatal, apenas alguns casos de histiocitose de células de Langerhans de idade tardia foram relatados. Lesões purpúreas que aparecem após o período neonatal podem sugerir histiocitose de células de Langerhans autolimitada e de início tardio. Uma vez confirmado o diagnóstico por biópsia cutânea, tais pacientes devem ser acompanhados regularmente, pois pode haver comprometimento sistêmico.

## INTRODUCTİON

Langerhans cell histiocytosis (LCH) is a generic term that identifies several clinical cases characterized by the proliferation of distinctive cells that are S100 and CD1a positive and contain Birbeck granules in their cytoplasm.^1^ ^,^ ^2^ “Self-healing” Langerhans cell histiocytosis (SHLCH) is a rare, self-limited variant of LCH that presents cutaneous lesions at birth or in the neonatal period with the absence of systemic manifestations and spontaneous resolution.^3^ Here we report a late-onset type of SHLCH, which was first diagnosed as isolated cutaneous LCH.

## CASE DESCRİPTİON

**Figure 1:**
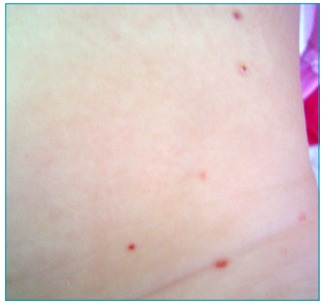
Erythematopurpuric and crusted papules on the torso

A 4½-month-old female patient presented to the pediatric dermatology clinic with an erythematopurpuric eruption on her torso. The parents reported that the the lesions had been present since she was 3-months-old. The patient was born at term after an uncomplicated pregnancy and was otherwise healthy with normal development for her age. Her physical examination was within normal limits and dermatologic examination revealed tiny erythematopurpuric papules, some of which were crusted, scattered over the torso (Fig. 1).

**Figure 2:**
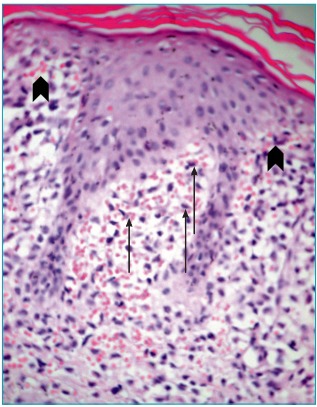
Dense infiltrate of neoplastic cells in papillary dermis (straight arrows) with sparse epidermal infiltration (arrowheads) accompanied by extravasated erythrocytes (hematoxylin and eosin; original magnification, X400)

**Figure 3:**
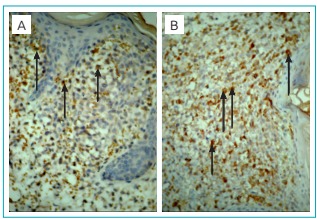
(A) Focal immunohistochemical staining with CD1a in Langerhans cells (arrows) (immunoperoxidase; original magnification, X400). (B) Diffuse and dense positive staining with S100 protein in the Langerhans cells (arrows) (immunoperoxidase; original magnification, X400).

An incisional biopsy was taken from one of the papules on the torso with clinical differential diagnoses of LCH and congenital leukemia cutis. The skin biopsy revealed a dense infiltrate of neoplastic cells in papillary dermis with sparse epidermal infiltration (Fig. 2). In an immunhistochemical analysis, the neoplastic cells were positive for S100 and CD1a and negative for mast cell tryptase, CD117, and myeloperoxidase (Fig. 3).

In a laboratory investigation, hemoglobin was 10.5 g/dL, total leukocyte count was 6,300/mm^3^, and platelet count was 285,000/mm^3^. Liver enzymes, renal function tests, serum chemistry, and urinanalysis were all within normal limits. Chest X-ray and skeletal radiographs did not show abnormalities. Ultrasonography of the abdomen and cranial magnetic resonance imaging were normal.

The patient was first diagnosed with isolated cutaneous LCH and no treatment was given to her. Within a few months, her lesions showed signs of regression. The patient is still being followed-up with and she is doing well. She has been without skin or systemic symptoms for four years, and now retrospectively evaluated as late-onset SHLCH.

## DİSCUSSİON

LCH represents a group of rare histiocytic disorders that are characterized by tissue infiltration with dendritic cells typically seen in infants and children. Three to four cases per million occur annually in children under 15 years of age with a peak incidence in infants aged one to two years old.^4^ ^,^ ^5^ The classification of histiocytic disorders are proposed by the World Health Organization as Class I (Langerhans cell histiocytosis), Class II (Histiocytosis of mononuclear phagocytes other than Langerhans cells, Familial and reactive hemophagocytic lymphohistiocytosis, Sinus histiocytosis with massive lymphadenopathy, Rosai-Dorfman disease, Juvenile xhantogranuloma, reticulohistiocytoma) and Class III (Malignant histiocytic disorders, Acute monocytic leukemia, malignant histiocytosis, True histiocytic lymphoma).^6^

The etiology of LCH is unknown, but neoplasia, immunostimulation, and dendritic cell disorders have been implicated in its pathogenesis.^7^ A common progenitor dendritic cell is hypothesized to give rise to Langerhans cells (LC) residing in the epidermis with dermal dendritic cells in the dermal and hypodermal areas.^8^

Congenital self-healing Langerhans cell histiocytosis (CSHLCH), also known as Hashimato Pritzker disease, is a rare, benign variant of histiocytosis. It is characterized by disseminated papules, vesicles, or nodules, occasionally with scaling, sometimes urticarial or hemangioma like. Affected infants are otherwise healthy and skin lesions tend to involute spontaneously within weeks to months.^9^ The diagnosis of LCH is based on histopathology, which is indistinguishable for all forms of LCH revealing a proliferation of CD1a and S100 protein-positive cells.^10^

After LCH is diagnosed, a thorough evaluation should be performed to rule out systemic involvement. The most common organs involved are the skin, liver, lymph nodes, bone marrow, spleen, and the skeletal system. A physical examination for lymphadenopathy, and an abdominal ultrasound for hepatosplenomegaly should be performed. A skeletal survey would reveal lesions within the skull or large bones. Urine osmalility should be checked to screen for diabetes insipidus.^10^

Neoplastic disorders to consider in a newborn with papulovesicles are congenital leukemia, LCH, and neuroblastoma.^11^ Characteristic histopathology and absence of other system involvement permit differentiation of benign forms of LCH. Because of the potential for recurrence in the skin or systematically, it has been suggested that the diagnosis of CSHLCH be made retrospectively, after a patient has remained free from systemic involvement for several years.^12^ About 100 cases of CSHLCH have been reported in the literature and we finally evaluated our case as CSHLCH after a four year follow-up. Regarding the age of onset, self-healing LCH is divided into the common type, which presents at birth or during the neonatal period, namely CSHLCH, and a late onset type, which presents after the neonatal period as observed in our case. Only a few cases of late onset SHLCH have been reported (Table 1).^11^ ^,^ ^13^ ^,^ ^14^ ^,^ ^15^ ^,^ ^16^ ^,^ ^17^ ^,^ ^18^

**Table 1:** Reported cases of late-onset Self-healing Langerhans cell histiocytosis.

Reference	Number of cases	Skin involvement	Age at onset
Nakahigashi et al.^11^	1	Multiple	8 years
Belhadjali et al.^13^	1	Multiple	20 days
Hashimoto et al.^14^	1	Multiple	17 days
Campourcy et al.^15^	1	Multiple	15 months
Jang et al.^16^	3	Multiple	1, 2, 7 months
Murata et al.^17^	1	Solitary	4 months
Nakahara et al.^18^	1	Multiple	10 months
Current patient	1	Multiple	3 months

CSHLCH generally carries a good prognosis. Its true incidence may be underestimated since spontaneous resolution often occurs before assessment by a dermatologist.^19^ CSHLCH patients with multisystem involvement may also show spontaneous regression.^20^ The self-regressing character of CSHLCH has been explained by the tumor cells of CSHLCH which eventually become apoptotic upon terminal maturation during the natural course of LC activation.^19^ However, there are no definitive clinical or histopathologic findings that reliably predict the long-term behavior of skin-only LCH in neonates; therefore, it is recommended that all patients be monitored at regular intervals throughout childhood with noninvasive monitoring.^10^ ^,^ ^21^ In neonates and young infants, cutaneous involvement is also the most common presentation of non-self regressive Langerhans cell histiocytosis (NSRLCH).^22^ Patients with systemic involvement may have a mortality rate as high as 20%. Also, it has been reported that of patients with LCH who initially presented with skin-only involvement at birth, 50% of cases had lesions that did not self-heal and later progressed to multisystem disease requiring treatment with systemic chemotherapy.^21^

However, the total mortality rate in infants initially diagnosed with CSHLCH in the literature is approximately 3%. The relatively substantial mortality rate in CSHLCH is noteworthy because CSHLCH has historically been considered a benign condition.^23^ While cutaneous involvement is observed in only 10% cases of children with single system LCH, the 53% incidence of cutaneous involvement is significantly higher in children with the multisystem disease.^21^ In the absence of systemic involvement, regular physical examinations for at least two years with repetition of blood work every six months is a valid approach in the long term management of patients with CSHLCH.^24^

There is no specific treatment for CSHLCH. Following the clinical picture and awaiting spontaneous regression is recommended. If the lesions persist, topical corticosteroids or topical nitrogen mustard may be effective. In cases of systemic recurrence, chemotherapy with vinblastine or etoposide, with or without corticosteroid is recommended.^3^ The early recognition of CSHLCH may spare children from redundant and potentially toxic systemic treatment.^9^ The most common sequela of CSHLCH is post-inflammatory hyper- or hypopigmentation.^23^

The course of LCH varies, from spontaneous resolution to a progressive multisystem disorder with organ dysfunction and potential life-threatening complications. Diagnosis of LCH is often difficult and may be delayed because of its rarity and especially so if it occurs with unusual presentation. A high index of suspicion and awareness of characteristic cytological features of LCH and its differential diagnoses is necessary.^25^ The late onset type of CSHLCH that appears after the neonatal period is a very rare and retrospective diagnosis. In the case of purpuric lesions, which serve as a clue for histiocytosis, the diagnosis should be confirmed by a cutaneous biopsy with immunohistochemical staining and the patients should be monitored at regular intervals to rule out systemic involvement.
